# Impact of Sociodemographic Characteristics on the Quality of Life of Frontline Nursing Staff During COVID-19 in Saudi Arabia

**DOI:** 10.7759/cureus.63263

**Published:** 2024-06-27

**Authors:** Muhammad B Maqsood, Azfar A Ishaqui, Shagufta Shaheen, Samia M Almutairi, Salman A Ahmad, Muhammad Imran, Zeeshan Ahmed, Javeria Farooq, Mohammad Al Arab, Zahida Akbar

**Affiliations:** 1 Department of Clinical Excellence, Eastern Health Cluster, Ministry of Health, Dammam, SAU; 2 Department of Pharmacy, Iqra University, Karachi, PAK; 3 Faculty of Humanities and Social Sciences, University of Central Punjab, Lahore, PAK; 4 Department of Health, Taif Directorate of Health Affairs, Ministry of Health, Taif, SAU; 5 Department of Clinical Research, Balsam Clinical Research (CR) - Phoenix Clinical Research, Riyadh, SAU; 6 Department of Obstetrics and Gynecology, King Abdulaziz Hospital, Ministry of National Guard Health Affairs, Al-Ahsa, SAU

**Keywords:** covid-19 pandemic, stress, well-being, sociodemographic characteristics, quality of life

## Abstract

Background

The COVID-19 pandemic imposed unprecedented challenges on healthcare systems worldwide. The pandemic placed frontline nursing staff working in the ICU and ER at the epicenter of this global crisis. This study aimed to assess the multifaceted impact of sociodemographic characteristics on the quality of life (QOL) of nursing staff during the pandemic.

Method

A cross-sectional survey was conducted to evaluate the impact of sociodemographic characteristics on the QOL of 322 frontline nurses working in the ICU and ER of five Saudi hospitals from May to July 2022. Participants completed the electronic survey questionnaire including demographic characteristics and four domains of QOL from the World Health Organization Quality of Life Questionnaire (WHOQOL-BREFF). The data was evaluated using descriptive and inferential statistics.

Results

Among 322 nurse participants, the majority were female (84.8%), married (64.4%), and held a bachelor's degree (92.4%). Age (above 40 years), gender (male), and marital status (married) reported a higher individual domain and overall QOL scores which shows that these characteristics have a direct influence on QOL. Years of work experience, extra working hours, and direct contact with COVID-19 patients were additional significant factors. Pearson correlation coefficients among QOL domains ranged from 0.54 to 0.91, indicating a strong interrelation among these domains. The highest transformed score was in the social domain (70.10) while the lowest score was in the psychological domain (59.20). The overall QOL mean score (SD) was 3.49(0.14) and the mean score (SD) of general health was 3.46(0.15).

Conclusion

The findings of this study suggest that sociodemographic and work-related factors have a complex and multifaceted impact on the QOL of nurses during the COVID-19 pandemic in Saudi Arabia. It also presents an insight into developing specific interventions to enhance nurses' resilience and well-being amidst pandemic challenges and to improve their QOL.

## Introduction

Working in the healthcare profession is both rewarding and challenging. Healthcare workers, particularly nurses in hospitals, face a great deal of stress in their jobs [1]. Nursing professionals may be contented with their efforts to improve patient outcomes but may be subjected to stress and burnout because of high workload and increased risk of exposure leading to turnover and adverse impact on patient care [2]. The quality of life (QOL) of nursing staff can be defined as the degree to which registered nurses may satisfy significant personal requirements via their experiences in their work organization while attaining the organization's goals [3].

Furthermore, nursing professionals treating COVID-19 patients may experience stress as a result of the work environment, lack of competence in infectious illness, and other factors [4]. According to the facts, nurses in most nations are under a lot of pressure at work. Workplace stress and burnout may result in a reduced QOL [5]. It is important to monitor and solve this problem since a lower quality of work life among healthcare workers might jeopardize patient care quality. Various research studies have identified that the majority of nurses found themselves unable to satisfy the patients' demands owing to staffing and financial constraints, leading to increased burnout and stress [6,7].

Healthcare crises like pandemics can increase the burden on healthcare workers in addition to their already hectic work schedule. Several attempts are made to evaluate the professionals' work-related QOL. Some researchers found that nurses have a moderate quality of work life, and some surveys found a significant degree of work discontent among nurses [8]. However, nurses may feel greater strain and stress when caring for COVID-19 patients [4].

It is imperative that owing to the COVID-19 pandemic, the QOL of nursing staff may deteriorate because of many reasons. Various research studies have identified that prolonged exposure to COVID patients [9], higher stress during the pandemic [10,11], reduced job satisfaction level [7,11], and extra shifts [12] have a direct impact on the quality of care provided to the patients by the nurses.

The impact of sociodemographic characteristics on the QOL of nurses during COVID-19 has been explored in several studies. A study conducted among nursing staff in Brazil reported that burnout negatively affects the QOL of nursing staff during the pandemic [13]. Psychological problems among nursing staff in Poland were not only limited to stress or bad emotions, but it was reported that depression, insomnia, and anxiety were very common during the pandemic COVID-19 [14].

Sociodemographic factors such as experience, age, education level, and marital status influence coping methods adopted by nurses during the pandemic [15]. A study conducted in Egypt found that nurses' awareness of COVID-19 infection and their QOL were related [16]. Nurses who improved their resilience were better equipped to cope with bad situations and had an increased ability to adapt and achieve [17]. The impact of sociodemographic characteristics on the QOL of nurses during COVID-19 was found to be significant, and healthcare institutions need to be prepared for pandemics with measures of psychological and social support for the protection and improvement of nurses' QOL [18]. In light of the challenges and stressors faced by nursing professionals, especially during the COVID-19 pandemic, understanding the socio-demographic factors that influence their QOL is crucial. The purpose of this study was to explore the impact of sociodemographic factors such as age, gender, marital status, and education on the QOL of nurses working in Saudi Arabian hospitals.

## Materials and methods

Study design

A cross-sectional survey-based multi-centered study investigated the QOL among hospital nurses during COVID-19. A validated survey questionnaire WHOQOL-BREF was used to collect data from respondents. This questionnaire is developed by the World Health Organization (WHO). This questionnaire has a high validity and reliability index [19]. The study was conducted from May to July 2021 in five Saudi Arabian hospitals situated in Riyadh, Jeddah, Medina, Dammam, and Al-Ahsa. The selected hospitals belong to the Ministry of National Guard Health Affairs. Riyadh is the most populated city in Saudi Arabia, situated in the central region. Jeddah and Madinah are ranked second and fourth respectively, in terms of population; both are in the Western region. Jeddah, Medina, and Dammam are the capital of their respective provinces.

Participants and eligibility criteria

The inclusion and exclusion criteria were defined before the study began. According to the criteria, participants were all licensed nursing staff working in the ICU and Emergency Room (ER). The length of service was decided to be more than one year to be included in the study. The eligible nursing staff were asked for their consent to participate in the study and upon agreement voluntarily, the participants were included in the study. All the nursing staff with less than a year of work experience were not part of the study because of insufficient work experience to clearly and impeccably differentiate how the quality of work life has been affected before and during the pandemic.

Sampling strategy and sample size

An online sample size calculator (RaoSoft Inc, Seattle, USA) was used to evaluate the sample size for the study [20]. There are around 4000 nurses involved in providing healthcare services to eligible patients of the Ministry of National Guard Health Authority (MNGHA) in five hospitals situated in Riyadh, Dammam, Medinah, Jeddah, and Al-Ahsa. This was taken as the target population and with 90% confidence interval and 5% marginal error with a 50% response distribution, the sample size was determined to be a minimum of 254 nurses. A consecutive sampling technique was used to collect responses.

All the communication related to the survey was carried out through an institutional electronic channel. The questionnaire and the ethics approval were emailed to the communication department of the organization. An online survey questionnaire form was created. The link to the questionnaire was emailed through the communication Services of MNGHA to all ER and ICU nursing staff working in the healthcare facilities.

Research instrument

The research instrument, WHOQO-BREF was used to evaluate the quality of work life among the nursing staff working in hospitals of Saudi Arabia. The WHOQOL-BREF research instrument comprises four domains containing items (questions) assessing the quality of life for that respective domain. These domains include physical health (seven items), psychological health (six items), social relationships (three items), and environmental health (eight items). Besides these four domains, the WHOQOL-BREF document also contains overall QOL and general health items [19]. Each item of the WHOQOL-BREF is scored from 1 to 5 on a response scale, which is stipulated as a five-point ordinal scale. The scores are then transformed linearly to a 0-100 scale [21,22].

Data analysis

An online survey form was used to collect the data in an MS Excel Spreadsheet (Microsoft Corporation, Redmond). The data entered in MS Excel was exported and analyzed using IBM SPSS Statistics for Windows, Version 26 (Released 2019; IBM Corp., Armonk, New York, United States). Descriptive statistics such as mean, standard deviation, and range were used to summarize the demographic characteristics. The t-test was applied to determine mean differences in QOL and general health groups across various participant characteristics. Pearson correlation revealed strong and statistically significant correlations between different QOL domains. Regression analysis was utilized to evaluate predictors affecting the four QOL domains, overall QOL, and general health. The models were adjusted for demographic variables like age, gender, and social status, as well as work-related and COVID-19-related variables. All models were rigorously evaluated for linearity, homoscedasticity, normality of residuals, and autocorrelation of residuals. Statistical significance was accepted at p<0.05.

Consent and ethics approval

An electronic version of the written informed consent was provided to all the participants before the start of the survey. Upon agreement, the online system directed them to the main survey form. The consent form contained the purpose of the study, voluntary participation, anonymity, and right to refusal from participation. The Institutional Review Board of King Abdullah International Research Center of King Saud Bin Abdulaziz University for Health Sciences granted ethical approval (RA20/012/A) for the study.

## Results

Initially, a total of 500 questionnaires were distributed by electronic mail. The response rate was only 50% in the first phase as there was a rise in COVID-19 cases and staff might be unable to respond. Then repeated reminders were sent and finally, a response rate of around 64% was achieved. Therefore sample of 322 nursing professionals was analyzed for this study. Most participants were female (N=273, 84.78%), married (N=206, 63.98%), had bachelor's degree (N=291, 90.37%), and were between 41 and 50 years old (N = 124, 38.50%). Most nursing professionals were non-Saudi (N = 288, 89.44%) and worked in hospitals of the Riyadh region (N=93, 28.88%). Most nurses had work experience between 10 and 15 years (N=88, 27.33%) and nearly two-thirds of nursing professionals (N=212, 65.83%) were not directly involved in caring for patients with COVID-19. The background characteristics are summarized in Table 1.

**Table 1:** Demographic and Professional Characteristics of Nursing Professionals in the Study (N=322)

Sociodemograhic Characteristics	Frequency	Percentage
Age		
20–30 years	40	12.42
31–40 years	73	22.67
41–50 years	124	38.51
51–60 years	79	24.53
>60 years	6	1.86
Gender		
Male	49	15.22
Female	273	84.78
Social Status		
Single	108	33.54
Married	206	63.98
Divorced	8	1.56
Educational qualiﬁcation		
Bachelors	291	90.37
Masters	25	7.76
Doctorate	6	2.5
Nationality		
Saudi	34	10.56
Non-Saudi	288	89.44
Region		
Riyadh	93	28.88
Jeddah	82	25.46
Al-Ahsa	65	20.18
Dammam	51	15.83
Medina	31	9.62
Work Experience		
Between 1 and 5 years	70	21.74
Between 5 and 10 years	65	20.19
Between 10 and 15 years	88	27.33
Between 15 and 20 years	48	14.91
20 years	51	15.84
Involved in direct care of patients with COVID-19		
No	212	65.83
Yes	110	34.16
Extra working hour		
No extra working hours	96	29.81
≤18 h (1–2 extra shift)	79	24.53
18 ≤ 36 h (3–4 extra shift)	83	25.77
36 ≤ 54 h (5–6 extra shift)	37	11.49
54 h (>7 extra shift)	27	8.38

The mean score of overall QOL is 3.49 (0.14), whereas the mean score for general health is 3.46 (0.15). The raw scores of each domain were transformed on a 0-100 scale where 0-20 means very poor QOL, 21-40 shows poor QOL, 41-60 indicates moderate QOL, 61-80 depicts good QOL, and 81-100 means very good QOL [19]. The findings show that the social domain has the highest transformed score of 70.10, while the environmental domain score was 66.25 and the physical domain score was 63.36. The lowest score was noted for the psychological domain 59.20. Table 2 elaborates on QOL scores across multiple domains and for overall QOL and health status.

**Table 2:** Quality of Life Scores (Overall QOL, General Health and Domains) QOL: Quality of Life; SD: Standard Deviation; Min: Minimum; Max: Maximum

Quality of Life Domains	Mean Score (SD)	Transformed Score (SD) 0-100	Range (Min-Max)
Overall QOL	3.49 (0.14)	-	1 - 5
General Health	3.46 (0.15)	-	1 – 5
Physical	24.81 (2.89)	63.36	7-35
Psychological	20.10 (2.39)	59.20	6-30
Social	11.41 (2.27)	70.10	3-15
Environmental	29.2 (3.09)	66.25	8-40

Transformed QOL scores in physical, psychological, social, and environmental domains, mean scores of overall QOL, and general health status across various sociodemographic factors are also interpreted. Age (above 40 years) is a significant determinant for physical, psychological, and social domains with higher mean QOL scores and p-value < 0.05. Gender has a statistically significant impact on physical, psychological, and environmental domains with higher mean scores for male with p-value < 0.05). Marital status influences all four domains of QOL; physical, psychological, social, and environmental, where married individuals had high scores with p-value < 0.05. Interestingly, education does not significantly affect any of QOL domains as indicated by p-value > 0.05 for all domains. These findings highlight the complex interplay of sociodemographic factors on the QOL of nursing staff. As far as other associated factors are concerned, years of work experience (> 15 years) is significantly associated with physical, psychological, and social domains along with overall QOL and general health with p-value < 0.05. Extra working hours are significantly related only to the physical domain with a higher score 67.91 and p-value=0.0008 along with higher scores of overall QOL (3.68, p-value=0.014) and general health (3.65, p-value=0.0.013), whereas no direct contact with COVID-19 patients has significant influence on physical, psychological, and environmental domains along with overall QOL and general health (p-value < 0.05). Nationality has no significant association with any QOL domain. The overall QOL score was highest for ages over 40 years (3.75) and the highest general health score was reported for males (3.78). These are given in Table 3.

**Table 3:** Transformed Scores across QOL Domains by Sociodemographic Factors QOL: Quality of life

Variables	Physical	Psychological	Social	Environment	Overall QOL	General Health
	Converted Score	Converted Score	Converted Score	Converted Score	Mean Score	Mean Score
Age (p-value)	< 0.0001	< 0.0001	0.0041	0.3317	< 0.0001	< 0.0011
< 40 years	60.48	63.26	71.83	64.26	3.18	3.29
> 40 years	71.84	73.12	75.81	72.67	3.75	3.67
Gender (p-value)	0.0096	0.0042	0.6107	0.0002	0.006	0.0032
Male	71.87	70.06	72.94	73.63	3.74	3.78
Female	62.64	57.22	69.75	64.28	3.45	3.49
Marital Status (p-value)	0.0125	0.0004	<0.0001	0.0057	< 0.0001	< 0.0010
Married	65.26	61.41	72.67	68.98	3.67	3.58
Single	60.15	56.17	66.89	59.03	3.34	3.38
Education (p-value)	0.8482	0.4234	0.5247	0.95	0.9821	0.9821
Bachelor's degree or higher	63.68	65.22	72.87	68.56	3.49	3.39
All Others	63.33	61.19	67.76	63.76	3.47	3.32
Work Experience (p-value)	0.0002	0.0001	0.0008	0.1004	0.0006	0.0260
< 15 years	58.87	57.11	65.51	59.33	3.29	3.39
> 15 years	67.91	67.01	72.55	69.47	3.64	3.61
Extra Working Hours (p-value)	0.0008	0.2327	0.5455	0.6002	0.0146	0.0134
No	69.71	62.14	72.88	70.45	3.68	3.65
Yes	52.01	54.31	59.47	59.82	3.28	3.32
Direct Contact with COVID-19 patients (p-value)	0.0341	0.0261	0.8905	0.0126	0.0292	0.0190
No	69.2	63.19	71.83	67.45	3.67	3.65
Yes	59.17	57.15	60.04	58.59	3.25	3.35
Nationality (p-value)	0.0841	0.0702	0.1703	0.8974	0.4023	0.2304
Saudi	59.41	57.54	75.48	67.82	3.45	3.33
Non-Saudi	63.72	55.35	71.56	64.71	3.42	3.27

Pearson correlation coefficients and p-values for the QOL domains, psychological, physical, social, and environmental domains, were analyzed. The correlation coefficients range from 0.54 to 0.91, all of which are statistically significant with p-values less than 0.0001. These findings indicate a strong and statistically significant correlation among the different quality of life domains. It also highlights that QOL domains are interlinked and may influence each other suggesting a holistic approach to improve the well-being of nurses. The strong correlations imply that targeted interventions to improve QOL in one domain may potentially benefit other domains, offering a more efficient use of resources and possibly lead to better job performance and patient care. Table 4 presents these findings.

**Table 4:** Pearson Correlation and p-Values across QOL Domains QOL: Quality of life

	QOL	Psychological	Physical	Social	Environmental
QOL	1.00000	0.85600	0.91026	0.75946	0.85194
		< .0001	< .0001	< .0001	< .0001
Psychological	0.85400	1.00000	0.74980	0.61669	0.53798
	< .0001		< .0001	< .0001	< .0001
Physical	0.91026	0.74980	< .0001	0.62037	0.71107
	< .0001	< .0001		< .0001	< .0001
Social	0.75946	0.61669	0.62037	1.00000	0.55822
	< .0001	< .0001	< .0001		< .0001
Environmental	0.85194	0.53798	0.71107	0.55822	1.00000
	< .0001	< .0001	< .0001	< .0001	

## Discussion

The current study explored the influence of various sociodemographic factors age, gender, marital status, and education on the QOL of frontline nursing staff during the COVID-19 pandemic. This research yielded several significant findings as it was conducted in multiple healthcare settings in Saudi Arabia. Most notably, age, gender, and marital status emerged as critical determinants of QOL among nurses where male, aged, and married individuals reported higher QOL scores. Conversely, education did not significantly impact QOL. These findings provide valuable insights into the complex interplay of sociodemographic factors affecting the well-being of nursing staff, particularly in the challenging context of a global pandemic.

This study's outcomes revealed that the QOL of nurses was significantly impacted during the COVID-19 pandemic and low scores in the psychological domain were probably owing to anxiety, stress, and despair. This aligns closely with the findings of a study that reported that stress, anxiety, and burnout were pervasive among healthcare workers and significantly affected their QOL. The study emphasized the emotional toll of caring for COVID-19 patients and the subsequent impact on healthcare workers' well-being [23]. However, our study diverges from the findings of another research that painted a direr picture of the psychological well-being of nurses. Their study reported that psychological problems among nurses were generally severe, with an overall incidence of mild to moderate distress at 28% and severe distress at 6%. This suggests that the psychological impact of the pandemic on nurses could be more severe than our study indicates, raising questions about the varying levels of resilience among healthcare workers in different settings [24].

Interestingly, our study also found elements of resilience and professional competency among nurses despite their challenges during the pandemic. This aspect of our findings resonates with a study arguing that having enough work experience during the COVID-19 pandemic represented both a traumatic event and an opportunity for personal and professional growth for nurses. This study also found that working extra hours during the pandemic only impacted the physical QOL domain probably owing to fatigue and exertion. However, it did not affect the other three domains which may indicate empathy and compassion of nursing staff for patients. The study highlighted that while posing significant challenges, the pandemic may offer healthcare workers a unique opportunity for skill development and emotional maturity [25].

This study found that age plays a significant role in determining the QOL among nurses during the COVID-19 pandemic. This is consistent with the findings of a study reported that nurses aged 41 years or older were more likely to take personal measures to cope with the pandemic [26]. However, another study suggested that age is associated with the subjectively perceived QOL among critically ill intensive-care patients [27]. This highlights the need for age-specific interventions in nurses e.g. separate behavioral and emotional training for young and older nurses to improve QOL.

The current study has highlighted that female nurses reported lower QOL scores. This is in line with a study's results that the vulnerability of nurses' health during COVID-19 was influenced by gender [28]. Another research study found that stress, anxiety, and burnout levels significantly affected the QOL of healthcare professionals, particularly females [23]. Our findings indicate that marital status has a distinct impact on QOL. Single nurses reported higher levels of stress as compared to married nurses. This might be due to family support and compassion, whereas it is contrary to the findings of a research study that reported no significant difference in the professional QOL of clinical nurses with respect to their marital status [29]. This phenomenon needs to be further explored to evaluate the impact of marital status and family support. Furthermore, in the current study educational level was not found significantly associated with QOL scores and nursing staff were holding bachelor's or higher degrees. This could be due to the assumption that the education level might be independent of knowledge, awareness, and readiness to deal with the COVID-19 pandemic. This aspect requires to be further explored in detail. Findings from a research study revealed that the majority of nurses held bachelor's degree and had an acceptable knowledge level of the COVID-19 pandemic while two-thirds were afraid of sharing the risk of exposure to the virus and half were stressed and overloaded [30]. 

In a nutshell, various sociodemographic variables have a significant impact on the QOL of nursing staff. Older nurses reported higher QOL scores, possibly due to greater experience and coping mechanisms developed over their careers. It could be explained that having ample experience may mitigate stress through better problem-solving skills and emotional regulation. Male nurses had higher QOL scores, which may be influenced by societal and cultural factors in Saudi Arabia that affect the distribution of responsibilities and being figurehead of the family. Married nurses reported higher QOL scores likely due to the emotional and social support from their families, which can buffer against work-related stress. The lack of significant impact of education on QOL suggests that practical experience and immediate work conditions may play a more critical role than formal education in determining nurses' well-being.

Our study has several limitations, including its cross-sectional design, sampling technique, and smaller sample size. These limitations may prevent researchers from making causal inferences. Additionally, the study was conducted in Saudi Arabia, limiting its generalizability to other cultural and healthcare contexts. Future research should aim to explore these variables longitudinally and in diverse settings.

## Conclusions

In conclusion, our study offers a comprehensive analysis of the QOL among frontline nursing staff during the COVID-19 pandemic by revealing a significant impact of various sociodemographic factors such as age, gender, marital status, and education. The findings corroborate and extend existing research, highlighting the complex interplay of these factors on nurses' QOL. It is also noteworthy that nurses reported resilience and professional growth despite the challenges posed by the pandemic. Some specific and actionable recommendations to improve nurses' QOL include enhancing the care experience and coping mechanisms in younger nurses, providing societal and cultural support to female nurses, giving emotional and behavioral counseling to manage work-related stress, and enhancing practical experience and work conditions to improve nurses' health and well-being.

We also recommend that healthcare administrators and policymakers consider implementing mental health support programs tailored to the needs of nurses particularly focusing on younger and female nurses, introducing flexible work schedules to help nurses balance professional and personal responsibilities, and providing targeted training and development programs to enhance coping skills and professional growth. Further research shall focus on exploring the longitudinal effects of the pandemic on nurses' QOL and evaluate the effectiveness of suggested interventions. Given the unprecedented challenges of the COVID-19 pandemic, a multi-faceted approach is crucial for enhancing the QOL of nursing staff which will ultimately improve the quality of healthcare services.
